# The dietary axis of endometriosis prevention: a gut microbiota-estrobolome framework situated within a multidimensional model of risk modification, early recognition, and disease management

**DOI:** 10.3389/frph.2026.1943184

**Published:** 2026-10-23

**Authors:** Sándor Nagy, Csaba Busznyák, Csilla Dézsi, Viktor Gulyás-Oldal, Ekaterina Paschali, Dániel Sallai, Imre Gálóczi, Károly Szili

**Affiliations:** 1 Department of Obstetrics and Gynecology, Faculty of Health and Sport Sciences, Széchenyi István University, Győr, Hungary; 2 Department of Obstetrics and Gynecology, Faculty of Medicine, University of Szeged, Szeged, Hungary; 3 Department of Molecular Biology, Faculty of Science, University of Szeged, Szeged, Hungary

**Keywords:** diet, early screening, endometriosis, estrobolome, faecal microbiota transplant, gut microbiota, prevention, probiotics

## Abstract

Endometriosis affects approximately 5%–10% of reproductive-aged women and imposes a substantial yet underexplored preventive research gap relative to treatment-focused literature. Diet is the central axis of this review: we organize the dietary evidence around an explicit mechanistic sequence—food-group-level epidemiological risk, hypothesised to be mediated by gut-microbiota dysbiosis and the estrogen-metabolizing “estrobolome”, and thence to candidate dietary and probiotic interventions—and then situate this dietary axis within a broader multidimensional prevention framework spanning additional primary prevention (other modifiable lifestyle factors, environmental endocrine-disruptor exposure), secondary prevention (non-invasive early screening, adolescent menstrual-health education, and, as a conceptual proposal, targeted early hormonal intervention following the neo-evolutionary model of Vercellini and colleagues), and tertiary-level management of established disease. While, to our knowledge, six recent reviews and meta-analyses (2024–2026) have quantitatively synthesized randomized-trial evidence on dietary and nutraceutical interventions for endometriosis-associated pain, none situates this evidence within an explicit gut microbiota-estrobolome mechanistic framework or a broader multidimensional prevention model—the distinguishing contribution of this review. We advance this framework as a hypothesis-generating prevention research agenda rather than a set of clinically established preventive measures. Cost-effectiveness is treated as a cross-cutting dimension: endometriosis-specific economic evaluations of prevention and screening remain scarce—with a digital symptom-checker Markov model as a notable exception—so analogous frameworks from chronic-disease lifestyle-intervention economics are proposed as methodological templates. We synthesize current evidence, controversies, and priority directions, with particular emphasis on the dietary axis and its mechanistic underpinnings.

## 1 Introduction

Endometriosis is a chronic, estrogen-dependent inflammatory condition defined by endometrial-like glandular and stromal tissue outside the uterine cavity. It affects an estimated 5%–10% of reproductive-age women worldwide, causing dysmenorrhea, deep dyspareunia, chronic pelvic pain, infertility and substantial quality-of-life loss. In the latest Global Burden of Disease analyses (1), prevalent cases among women of childbearing age rose from approximately 19.87 million in 1990 to 22.28 million in 2021—about 12%. This rise reflects population growth, not rising age-standardized rates, which declined modestly (estimated annual percentage change −0.98% for prevalence). Attributable disability-adjusted life-years rose in parallel from approximately 1.83–2.05 million, and are projected to keep rising toward 2050 as the female reproductive-age population grows. Incidence peaks at 20–24 years and prevalence at 25–29 years, placing peak burden within the reproductive and often family-building years. Approximately 26% of women diagnosed with endometriosis also experience infertility (1)—a proportion of the diagnosed, not general, population—roughly four-fold the rate in unaffected women.

Diagnostic delay remains a defining feature of the disease trajectory. Multiple health-system studies report an average delay of 4–11 years between symptom onset and definitive diagnosis, varying by country and health system (2, 3). This lag prolongs symptoms and increases healthcare use, through repeated, often unfocused, contacts before the correct pathway is engaged; its effect on lesion progression is uncertain, because progression is not inevitable and no prospective study has shown that earlier intervention alters the natural history of endometriosis.

Most of the endometriosis research portfolio addresses treatment—hormonal suppression, surgical excision, and assisted reproduction—rather than prevention, an asymmetry recognized as a structural gap. Vercellini and colleagues have proposed a “neo-evolutionary-oriented” secondary prevention framework. They argue that early-onset endometriosis and adenomyosis share a pathogenic trajectory that is, at least in principle, interceptable before irreversible fibrotic and neuro-inflammatory changes accumulate (4, 5). Their two-part proposal—pathogenic rationale in Part I, candidate medical interventions in Part II—anchors this review but is deliberately not diet-centered and does not address cost-effectiveness.

This review extends that framework along three axes that must be considered jointly, not in isolation. First, primary prevention encompasses modifiable lifestyle and dietary exposures and environmental endocrine-disrupting chemicals. Second, secondary prevention encompasses non-invasive early screening tools and adolescent menstrual-health education. Third, tertiary prevention encompasses managing established disease and attenuating progression, irreversible sequelae and recurrence. Within the second, following its own authors, we include targeted early interception of newly identified early-onset disease. A cross-cutting fourth dimension—cost-effectiveness—applies throughout, since a prevention strategy's policy relevance depends on economic and clinical justification.

Within this structure, diet receives emphasis. Previous dietary syntheses have treated nutrition as a symptom-management adjunct assessed through randomized-controlled-trial (RCT) outcome counting. Unlike them, we organize the dietary evidence around an explicit mechanistic axis, running from food-group-level epidemiological associations with diagnosis risk, hypothesized to be mediated by gut microbiota dysbiosis and estrogen-metabolizing (“estrobolome”) pathways, to candidate dietary and probiotic interventions. We emphasize that the axis's mediating steps are hypothesized rather than demonstrated in humans. None of the interventions discussed is currently ready for clinical use as a preventive measure. Six reviews and meta-analyses published between 2024 and 2026 have quantitatively synthesized the RCT evidence for dietary and nutraceutical interventions in endometriosis. These comprise Meneghetti et al. (6) and Wójtowicz et al. (7), plus four narrower reviews addressing antioxidants, anti-inflammatory supplements, and vitamin C/E. Not all are systematic reviews or meta-analyses; several are critical or narrative reviews. We do not duplicate that synthesis; Section 3.6 sets out how this review is positioned relative to it.

The current review is organized around the framework's four dimensions. Section 2 covers modifiable lifestyle factors other than diet. Section 3 presents the dietary axis: risk-factor epidemiology (3.1), the gut microbiota-estrobolome mechanistic bridge (3.2), micronutrient supplementation (3.3) and therapeutic dietary protocols (3.4). It then turns to probiotics (3.5), the recent randomized-trial syntheses (3.6), and the microbiome-directed research frontier (3.7). Section 4 addresses endocrine-disrupting chemicals; Section 5, earlier non-invasive detection; Section 6, early hormonal interception; Section 7, cost-effectiveness; and Section 8 draws out controversies, gaps and clinical implications. Figure 1 summarizes the model developed across Sections 2–7.

**Figure 1:**
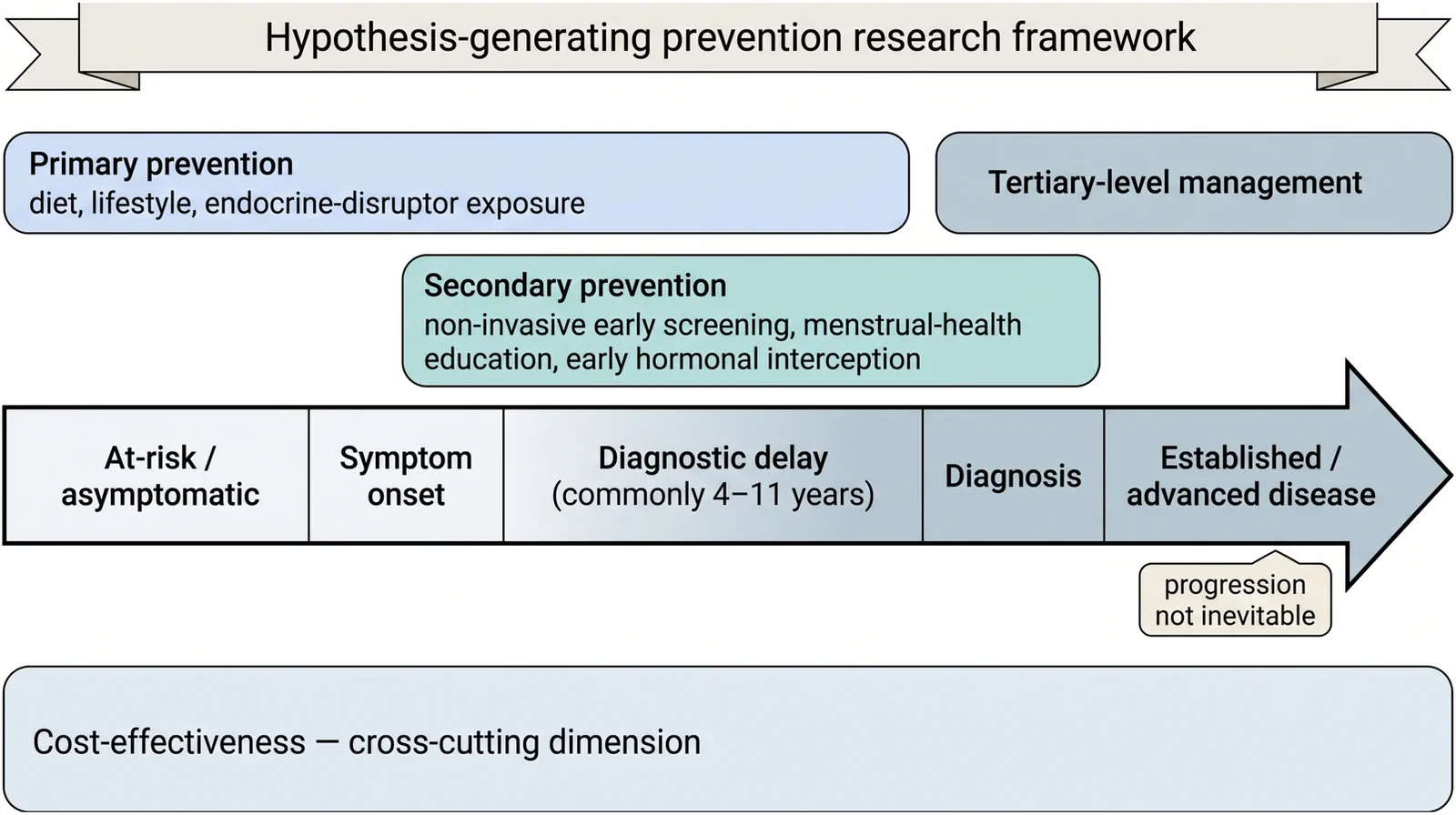
A four-dimensional prevention framework for endometriosis. Primary, secondary and tertiary prevention tiers are positioned along the disease trajectory according to the stage at which each acts, with cost-effectiveness applied as a cross-cutting dimension across all tiers. Early hormonal interception (Section 6) sits within the secondary tier because it couples early detection with early intervention. The shaded segment denotes the interval between symptom onset and diagnosis (commonly 4–11 years) (2, 3), the principal target of secondary prevention. The trajectory is drawn as a schematic sequence and not as an inevitable course: progression to advanced disease is not universal, symptom severity correlates only imperfectly with lesion burden, and some lesions remain stable or regress—hence the “not inevitable” annotation at the right-hand endpoint. The framework as a whole is hypothesis-generating and is not a validated clinical pathway. EDC, endocrine-disrupting chemical; ICER, incremental cost-effectiveness ratio; QALY, quality-adjusted life-year. Created in BioRender. Szili, K. (2026) https://BioRender.com/pni26ct.

### 1.1 Search strategy and scope

This is a narrative review; it does not follow PRISMA and no protocol was registered. We searched PubMed/MEDLINE, Scopus and the Cochrane Library from database inception to 30 May 2026, restricted to English-language, peer-reviewed, journal-indexed publications. We combined endometriosis with diet, dietary pattern, gut microbiota/microbiome, estrobolome, β-glucuronidase, probiotic, fecal microbiota transplantation, FODMAP, gluten, physical activity, endocrine-disrupting chemicals, screening, diagnostic delay and cost-effectiveness. A representative string was: [endometriosis(Title/Abstract)] AND (diet OR “dietary pattern” OR “gut microbiota” OR microbiome OR estrobolome OR probiotic OR FODMAP OR gluten)[Title/Abstract], with equivalent syntax in Scopus and Cochrane. Reference lists of retrieved reviews were hand-searched. Records were eligible if they reported original data, a systematic synthesis, an economic evaluation or a mechanistic model bearing on endometriosis risk, detection, progression or symptom management. Conference abstracts, preprints, non-peer-reviewed sources and articles without accessible full text were excluded. Two authors screened and extracted data independently, resolving disagreements by discussion among K.Sz., V.G.-O. and Cs.D. Because the search was not systematic, all novelty and exclusivity statements are phrased “to our knowledge”. For quantitative randomized-trial evidence on dietary and nutraceutical interventions, this review relies on six recent syntheses (2024–2026) rather than repeating their primary-trial screening, stated explicitly. These six were identified by the same search, limited to reviews or meta-analyses of dietary, nutraceutical or supplement interventions in endometriosis from 2024 onward. Where they overlap in primary trials, we cite the most recent, most complete synthesis and note the overlap rather than counting a trial twice. Literature appearing during manuscript preparation was added only where it bore directly on a claim already made. Landmark primary studies predating 2022 (e.g., large prospective cohorts and the sole controlled dietary-intervention trials) are retained where no more recent equivalent primary evidence exists. Reference metadata (journal, DOI, indexing status, and publication age) were verified against Crossref and NCBI. The reference-validation table (Supplementary Table S1) and reference file (Supplementary File S2) document this check.

### 1.2 Defining prevention in this review

The interventions considered act at different points on the disease course. We therefore classify each exposure and intervention by two criteria—the population studied (at-risk or asymptomatic vs. already-diagnosed) and the outcome measured—assigning it to one of three operational tiers. Primary prevention denotes reducing incident disease before lesions establish, in at-risk or general populations. Within this tier we distinguish (a) prevention of initial lesion establishment from (b) modification of the factors associated with receiving a diagnosis, because most dietary epidemiology measures the latter rather than lesion incidence. Secondary prevention denotes earlier detection and early intervention to shorten the diagnostic interval and intercept early progression. Tertiary prevention denotes managing established disease, including attenuating progression, irreversible sequelae and recurrence, and controlling symptoms.

Applying this scheme, observational diet–diagnosis associations constitute primary-prevention (diagnosis-risk) evidence. Low-FODMAP, probiotic and most nutraceutical trials are conducted in already-diagnosed patients and measure symptom or quality-of-life endpoints; they constitute tertiary-level management, not evidence for primary prevention. Non-invasive biomarkers, symptom-checker triage and adolescent menstrual-health education are secondary-prevention tools. The Vercellini neo-evolutionary model of early hormonal interception, coupling early detection with early intervention, is a secondary-prevention proposal. Table 1 classifies every exposure and intervention, and Table 2 summarizes the GRADE certainty of principal conclusions.

**Table 1:** Classification of exposures and interventions by the prevention tier to which the evidence genuinely applies.

Exposure/intervention	Population & disease status	Design (*n*)	Comparator	Outcome measured	Direction & effect (as reported)	Certainty (GRADE-informed)	Prevention tier	Evidence type
Vegetables (higher intake)	At-risk/general women	Cohort + case-control, pooled [Arab (16); Neri (17)]	Low vs. high	Endometriosis diagnosis	Lower diagnosis risk (RR = 0.59)	Low	Primary (diagnosis-risk)	Observational
Fruit (higher intake)	At-risk women	Cohort/case-control [Arab (16)]	Low vs. high	Diagnosis	Mixed/lower	Very low	Primary (diagnosis-risk)	Observational
Dairy/cheese (higher intake)	At-risk women	Cohort + dose-response MA [Qi (24)]	Low vs. high	Diagnosis	Lower (cheese OR 0.84; dairy RR 0.87)	Low	Primary (diagnosis-risk)	Observational
Red meat (higher intake)	At-risk women	Prospective cohort [Yamamoto (19)]	Low vs. high	Diagnosis	Higher risk	Low	Primary (diagnosis-risk)	Observational
Trans-unsaturated fat	At-risk women	Prospective cohort [Missmer (18)]	Q1 vs. Q5	Diagnosis	Higher risk (RR 1.48, CI 1.17–1.88)	Low	Primary (diagnosis-risk)	Observational
Omega-3 (dietary intake)	At-risk women	Prospective cohort [Missmer (18)]	Low vs. high	Diagnosis	Lower/mixed	Very low	Primary (diagnosis-risk)	Observational
Alcohol	At-risk women	MA of cohort/case-control [Li Piani (14)]	Non-drinkers	Diagnosis	Higher (modest)	Low	Primary (diagnosis-risk)	Observational
Mediterranean/anti-inflammatory pattern	Confirmed disease	Prospective single-arm [Cirillo (22), *n* = 35]	Baseline	Pain, oxidative stress	Lower pain; lower oxidative stress	Very low	Tertiary (symptom management)	Uncontrolled
Physical activity	At-risk (adolescent window)	Cohort [Vitonis (9)]; Garavaglia (12) + SR/MA [Ricci (10))	Low vs. high	Diagnosis	Inconsistent/lower	Very low	Primary	Observational
Smoking	At-risk women	Cohort/MA [Li Piani (14)]	Non-smokers	Diagnosis	Inconsistent	Very low	Primary (diagnosis-risk)	Observational
Endocrine-disrupting-chemical reduction	Pre-clinical/at-risk	Mechanistic + observational [Wieczorek (67); Dutta (68)]	Higher vs. lower	Incidence	Hypothesised lower	Very low	Primary (lesion incidence)	Mechanistic
Vitamin D supplementation	Diagnosed	RCT [SAGE, Nodler (34)]	Placebo	Pain	Null	Low	Tertiary (symptom management)	RCT
Omega-3 supplementation	Diagnosed	RCT [SAGE, Nodler (34); PurFECT1, Abokhrais (39)]	Placebo	Pain	Small/inconsistent	Low	Tertiary (symptom management)	RCT
Low-FODMAP diet	Diagnosed ( ± IBS)	Uncontrolled/observational [van Haaps (42); De Araugo (43)]	Baseline	GI symptoms, QoL	Improved GI symptoms (not lesion-modifying)	Very low	Tertiary (symptom management)	Observational
Gluten-free diet	Diagnosed	Single-arm pre–post [Marziali (46)]	None	Pain	Improved [uncontrolled; Becker (91): insufficient evidence]	Very low	Tertiary (symptom management)	Uncontrolled
Probiotic (oral Lactobacillus)	Stage III–IV confirmed [Khodaverdi (49), *n* = 37]	RCT, placebo	Placebo	Pain severity	Reduced pain	Low	Tertiary (symptom management)	RCT (small)
Postbiotic (heat-killed L. gasseri)	Clinically diagnosed [Itoh (50)]	Controlled trial	Placebo	Dysmenorrhoea/pain	Reduced	Very low	Tertiary (symptom management)	Trial (small)
Faecal microbiota transplantation	Rodent models only	Preclinical, mouse [Xu (32)]	Donor vs. endo FMT	Lesion volume	Reduced = 4-fold (rodent)	Very low (indirect)	Experimental (no tier)	Animal
Non-invasive biomarkers (miRNA, CA-125)	Symptomatic women	Diagnostic-accuracy studies [Li (71); Irungu (72); reviews to 2025]	Laparoscopy (reference)	Detection (AUC)	Discriminative in discovery cohorts; not externally validated	Low	Secondary (early detection)	Diagnostic
Digital symptom-checker	Symptomatic (direct-to-patient)	Validation/economic model [Xu (75)]	—	Triage/time-to-diagnosis	Modelled benefit	Very low	Secondary (early detection)	Modelling
Adolescent menstrual-health education	Adolescents	Pre-post program [Bush (79) + cluster-RCT (Wahl (84)]	None	Symptom recognition/referral	Improved recognition	Very low	Secondary (early recognition)	Uncontrolled
Early hormonal interception (Vercellini)	Confirmed early-stage adolescents/young women	Conceptual proposal [Vercellini (4, 5)]	—	Progression (unproven)	Hypothesised prevention of progression	Very low	Secondary (early intervention)	Conceptual

GRADE certainty and the population/outcome basis for each assignment are shown; effect estimates are as reported in the cited sources. The certainty column applies an adapted, GRADE-informed judgement to each individual exposure or intervention rather than a formal GRADE assessment of a body of evidence for a specific outcome; formal GRADE ratings for the review's principal conclusions are given in Table 2. GRADE certainty was rated from study design and rated down for risk of bias, inconsistency, indirectness, imprecision and publication bias: randomized trials start at high certainty and observational studies at low, and evidence that is purely mechanistic or animal-derived is rated down further for indirectness. Prevention tiers: primary (pre-disease risk reduction, distinguishing lesion incidence from diagnosis risk), secondary (early detection and early intervention), tertiary (management of established disease and its complications). The publication year of the principal source is given in the design column and the evidence type in the final column, so that established findings can be distinguished from emerging 2025–2026 literature; an em dash denotes not applicable.

**Table 2:** Summary of the principal conclusions with their GRADE certainty of evidence.

Key conclusion	GRADE certainty	Basis for rating
No specific diet is established to prevent incident endometriosis	Very low	Observational; ascertainment bias; no incidence RCTs
Higher vegetable/fibre intake is associated with lower risk of a diagnosis	Low	Repeated observational associations; residual confounding and ascertainment bias
Trans-fat intake is associated with higher risk of a diagnosis [Missmer (18)]	Low	Single large prospective cohort [Missmer (18)]; observational
Low-FODMAP diet improves GI symptoms, not endometriotic disease	Low (GI outcome)	Uncontrolled; indirect for disease
Routine gluten-free diet is not supported without another indication	Very low	Uncontrolled pre-post; Becker et al. (91) (ESHRE): insufficient evidence
Vitamin D/omega-3 supplementation shows no consistent pain benefit	Low	Small RCTs incl. null SAGE trial
Probiotics are an early clinical signal requiring confirmation	Low/Very low	Two small, heterogeneous trials; strain-specific
No controlled human endometriosis FMT data exist	Very low	Rodent models only; high indirectness
The gut microbiota–estrobolome causal pathway is a hypothesis	Very low	Most human evidence compositional; downstream extrapolated
Whether early hormonal interception modifies long-term progression is unresolved	Very low	No prospective validation; conceptual

Certainty ratings follow the criteria stated in the legend to Table 1; the basis column names the specific reason for the rating in each case.

## 2 Primary prevention I: modifiable lifestyle factors

Beyond diet, several modifiable lifestyle exposures have been associated with endometriosis risk in prospective cohorts. Hemmert et al. (8), using the Utah Population Database and clinical cohort data, identified a cluster of modifiable lifestyle factors—including body composition, alcohol use, and physical activity—associated with incident endometriosis risk. The authors caution, however, that reverse causation, whereby undiagnosed early-stage disease influences behavior, cannot be fully excluded in observational designs.

Physical activity has received the most consistent attention. In the Nurses' Health Study II, a large prospective cohort, Vitonis et al. (9) reported that higher adult physical activity was associated with a modestly reduced risk of endometriosis diagnosis, most apparent for vigorous activity sustained from adolescence into adulthood. A subsequent systematic review and meta-analysis by Ricci et al. (10), pooling observational studies in women with infertility or pelvic pain, similarly reported a protective association between physical activity and endometriosis risk. It noted substantial heterogeneity in how “activity” was operationalized, as frequency, intensity, and life-stage of exposure all varied. A complementary systematic review focusing specifically on exercise interventions rather than habitual activity reached more equivocal conclusions, reflecting the paucity of interventional, as opposed to observational, data (11).

Mechanistically, several pathways may link physical activity to reduced endometriosis risk: reduced circulating estrogen via decreased aromatization in adipose tissue, anti-inflammatory effects mediated by myokine release, and improved retrograde-menstruation clearance via enhanced pelvic and peritoneal lymphatic flow. Direct mechanistic confirmation in human tissue remains limited. Occupational and leisure-time activity at different life stages may carry distinct risk profiles (12). Some evidence suggests that activity during adolescence, a plausible critical window for early lesion establishment, may be more protective than that initiated later in reproductive life. This is consistent with the broader “early-life exposure” literature linking in-utero and peripubertal factors to later endometriosis risk (13).

Alcohol has attracted attention because ethanol raises circulating endogenous estrogen, a plausible mechanism for an estrogen-dependent disease. An initial meta-analysis to 2012 reported a positive association with endometriosis risk. An updated synthesis of 23 studies published through October 2021 found it attenuated to borderline significance (unadjusted odds ratio 1.14, 95% CI 0.99–1.31, *p* = 0.06) (14). This shows how an initially significant epidemiological signal weakens as further, sometimes methodologically stronger, studies accumulate. Cohorts within that synthesis diverged: one followed 473 women and found no association; another, of 28,882 women, reported positive associations for smoking and coffee but not alcohol. Smoking and possibly caffeine are therefore more consistently implicated than alcohol. However, none of these associations approaches an effect size justifying population-wide behavior-change messaging alone. Each belongs in a broader lifestyle counseling package rather than a specific preventive recommendation.

The lifestyle evidence supports physical activity as a plausible, low-cost primary-prevention lever. Yet nearly all data are observational, hence vulnerable to reverse causation and residual confounding. Lifestyle counseling should therefore be framed as a reasonable adjunct, not a proven risk-reduction strategy, pending interventional confirmation.

One methodological theme recurs in Section 3.1: exposures may carry disproportionate weight during a critical developmental window. Physical activity matters in adolescence, alcohol and smoking in the perimenarchal years. Studies measuring exposure only in adulthood may therefore underestimate any causal effect. Life-course designs capture exposure timing, not only its presence or intensity. Applied only sporadically here, such designs would materially strengthen this literature.

## 3 The dietary axis across risk reduction and disease management

### 3.1 Risk-diet epidemiology

Four features of this literature bound what the estimates below can mean; we state them here rather than in the Discussion. First, dietary exposure is almost always self-reported, usually by food-frequency questionnaire, and thus subject to substantial measurement error. Non-differential error generally attenuates associations, whereas differential recall in case-control designs can inflate them. Second, diet may change in response to symptoms rather than the reverse. Women with undiagnosed endometriosis frequently have gastrointestinal symptoms and report altering their diet accordingly (15). Reverse causation therefore cannot be excluded from any cross-sectional or case-control estimate. Third, and most consequentially, the outcome measured is not lesion incidence but receipt of a clinical diagnosis. That diagnosis depends on healthcare access, referral patterns, clinician index of suspicion and historical laparoscopy availability. Women with healthier diets also differ in socioeconomic position, physical activity and healthcare engagement. Some part of any observed association may therefore reflect who is investigated rather than who develops disease. We accordingly describe exposures as associated with a lower or higher risk of receiving a diagnosis, not as protective or harmful. Fourth, the estimates are not commensurable. Odds ratios from case-control studies and relative risks from prospective cohorts are different quantities, and case definitions range from surgically confirmed disease to self-report. Pooling them—as the umbrella and meta-analytic sources below necessarily do—introduces heterogeneity that the pooled confidence intervals do not capture. For these reasons this evidence base is rated low or very low certainty (Table 1).

The most comprehensive synthesis of dietary risk-factor epidemiology is the systematic review and meta-analysis of Arab et al. (16), pooling cohort, case-control and cross-sectional studies of food-group and nutrient intake and endometriosis risk. A subsequent umbrella review by Neri et al. (17) gives the most precise estimates available. Registered in PROSPERO, it synthesized ten primary systematic reviews by Joanna Briggs Institute methodology. Diagnosis risk was lower with higher vegetable intake (relative risk 0.59, 95% CI 0.49–0.71, *p* < 0.001). It was also lower with cheese (odds ratio 0.84, 95% CI 0.74–0.96) and total dairy (relative risk 0.87, 95% CI 0.81–0.95). All were classified as low-certainty (class IV) evidence. We deliberately do not restate these as percentage risk reductions. A relative risk of 0.59 describes an association with the probability of a diagnosis, in low-certainty observational data with the confounding structure just described. Rendering it as a “41% reduction” would imply an individual-level preventive effect the design cannot support. A protective point estimate was also reported for high-fat dairy. Its published confidence interval, however, is internally inconsistent with the point estimate, and warrants particular caution pending verification against the underlying primary studies.

The dietary-fat evidence warrants careful statement because it is frequently misreported. The single most informative primary study is Missmer et al.'s prospective analysis (2010; 70,709 women, Nurses' Health Study II). There, higher trans-unsaturated fat intake was associated with increased, not decreased, risk of laparoscopically confirmed endometriosis (highest vs. lowest quintile relative risk 1.48, 95% CI 1.17–1.88, p-trend 0.001). Higher long-chain omega-3 intake, by contrast, carried lower risk. Isolated opposite findings exist within the umbrella review, but this substantially larger, more rigorously designed cohort contradicts them; they should not be the primary evidence. Butter specifically has been associated with increased risk (relative risk 1.27). This heterogeneity across animal-fat food categories—lower risk with total dairy and cheese, higher with butter and trans-fat—illustrates why food-group associations should not be over-interpreted as evidence for a single unifying inflammatory-fat mechanism. It probably reflects undisentangled food-matrix effects (dairy's calcium, vitamin D fortification and bioactive peptides potentially offsetting its saturated fat).

In absolute terms, endometriosis remained uncommon in these cohorts. Missmer et al. (18) recorded 1,199 laparoscopically confirmed incident cases over 586,153 person-years, an incidence of approximately 2 per 1,000 person-years. Against that baseline, a relative risk of 1.48 corresponds to an absolute difference of well under one additional confirmed case per 1,000 women per year. This baseline is our derivation from the reported overall rate, not a figure given in the source. That difference counters the impression that relative risks of this size denote a large individual-level effect. The prospective gluten analysis in Section 3.4 is similarly informative on scale, with 3,810 confirmed cases among 81,961 women over 24 years. The remaining dietary studies report only relative measures; per-food-category absolute rates were not recoverable from the source reports. Where not recoverable, we say so rather than estimate.

A large prospective cohort analysis by Yamamoto et al. (19), nested within Nurses' Health Study II, examined meat and fish consumption specifically. Higher red meat intake was associated with increased endometriosis risk, whereas some seafood/fish patterns showed weaker or null associations. This is broadly consistent with an inflammatory-mediator hypothesis (red meat supplying arachidonic-acid-derived pro-inflammatory eicosanoid precursors) rather than a single unifying nutrient explanation. An earlier population-based case-control study by Trabert et al. (20) reported broadly concordant directional findings for several food groups, reinforcing the red-meat-risk association across independent designs. Parazzini et al. (21), in an earlier narrative synthesis, flagged the directional consistency of red-meat-risk and vegetable/fruit-protection associations across the then-available cohort and case-control literature. They cautioned, however, that confounding by socioeconomic status, healthcare-seeking behavior and diagnostic access could not be excluded.

Beyond individual food groups, whole dietary-pattern analyses offer a complementary approach less vulnerable to single-nutrient confounding. To our knowledge, no case-control study has yet quantified Mediterranean, MIND or Alternative Healthy Eating Index adherence against incident endometriosis; we make no such claim. Mechanistically, a separate analysis linked Mediterranean-diet adherence to reduced oxidative-stress biomarkers and lower pain-perception scores in women with confirmed endometriosis (22). It offers a plausible intermediate pathway (antioxidant and polyphenol density) connecting whole-pattern dietary quality to symptom burden, complementing the food-group and single-nutrient literature above. A further review of dietary patterns and supplementation in endometriosis management concluded similarly: pattern-level dietary quality, not any single excluded or supplemented nutrient, may carry the more mechanistically coherent signal (23). This matters when counseling patients, who are often presented, particularly through non-peer-reviewed sources, with many single-nutrient restriction recommendations of uncertain value. A systematic review and dose-response meta-analysis of dairy intake (24) reported the modestly protective association already noted. It adds a further food-group data point to the broader animal-product heterogeneity that complicates any single “avoid animal products” public-health message.

### 3.2 Gut microbiota-estrobolome mechanistic bridge

The mechanistic link between diet and endometriosis risk/progression is increasingly framed through gut microbiota, specifically the “estrobolome”. This is the aggregate of gut-bacterial genes and enzymes, principally β-glucuronidases, that deconjugate hepatically conjugated estrogens back into their biologically active form within the enterohepatic circulation. Deconjugated estrogen is reabsorbed rather than excreted, increasing systemic and peritoneal estrogen exposure. This is a plausible amplifying mechanism for an estrogen-dependent disease such as endometriosis.

A 2025 systematic review and meta-analysis pooled eleven studies (1,727 women, including 433 with diagnosed endometriosis and 1,294 controls) (26). It reported consistent gut microbial alpha- and beta-diversity differences between women with and without endometriosis. Its authors emphasize that cross-sectional case-control designs cannot establish whether dysbiosis is causal, consequential, or bidirectionally reinforcing for lesion establishment. Guo and Zhang (25), in a narrative synthesis, integrate this diversity evidence with proposed causal pathways. Gut dysbiosis is hypothesized to increase intestinal permeability, permitting bacterial lipopolysaccharide (LPS) translocation into systemic circulation. This activates the Toll-like receptor 4 (TLR4)-nuclear factor-κB (NF-κB) inflammatory signaling axis implicated in lesion establishment and pain sensitization. Complementary narrative reviews integrate this microbiome axis alongside the more established hormonal and immune (natural-killer-cell dysfunction, macrophage-mediated peritoneal inflammation) mechanisms. They situate diet-microbiome interaction as one node within a broader multi-hit pathogenic model rather than a standalone causal pathway (27). Most mechanistic steps in this bridge rest primarily on evidence from other chronic inflammatory conditions and animal models. These steps—β-glucuronidase-mediated estrogen deconjugation, LPS/TLR4-NF-κB signaling, short-chain-fatty-acid effects, and gut-brain pain modulation—have not been directly demonstrated in human endometriotic tissue. The estrobolome framework should therefore be read as a biologically plausible, testable organizing hypothesis rather than an established causal pathway.

This bridge is directly relevant to prioritizing which future interventions merit dedicated trials (e.g., probiotic strains selected for β-glucuronidase-inhibiting activity, vs. generic multi-strain probiotics). We stress that this sequence is a graded hypothesis rather than an established mechanism, and that the human evidence sits almost entirely at its first rung. The evidence ladder ascends through five rungs: (i) compositional case-control differences; (ii) functional or enzymatic change (gut β-glucuronidase activity); and (iii) a demonstrable shift in circulating or tissue estrogen. The higher rungs are (iv) a measurable effect on human endometriotic lesions; and (v) evidence that modifying the microbiome changes clinical outcomes. In women with endometriosis, rungs (ii) to (v) remain largely undemonstrated. Accordingly, β-glucuronidase-targeting probiotic strains are best regarded as investigational rather than clinically ready.

Beyond the estrobolome mechanism, the broader gut-microbiome literature proposes at least two additional, non-mutually-exclusive pathways. First, short-chain fatty acids (SCFAs, principally butyrate, propionate and acetate) are produced by bacterial fermentation of dietary fiber. They have documented anti-inflammatory and regulatory-T-cell-promoting effects in other chronic inflammatory conditions. They are also hypothesized to counteract the pro-inflammatory, NF-κB-driven signaling implicated in lesion maintenance and pain sensitization, though this remains undemonstrated in endometriosis-specific human tissue. This pathway would predict that fiber-rich dietary patterns act partly via microbiome-mediated SCFA production rather than direct nutrient effects alone. Such patterns, broadly consistent with the vegetable-protective and Mediterranean-pattern associations in Section 3.1, offer one plausible unifying mechanism connecting the food-group epidemiology of Section 3.1 to the microbiome mechanism here (28). Second, gut dysbiosis has been proposed to influence peripheral and central pain processing directly via the gut-brain axis (vagal afferent signaling and microbiome-derived neuroactive metabolites). This would be relevant to endometriosis-associated central sensitization and comorbid conditions such as irritable-bowel-syndrome-like symptoms, reported at elevated frequency in endometriosis patients. Several dietary intervention studies in Section 3.4 (particularly the low-FODMAP trials) appear to target them as much as, or more than, the underlying gynecological pathology (29).

The human microbiome case-control literature here remains early and primarily descriptive. Cross-sectional composition differences, even when robust and replicated, cannot establish the direction of the relationship. They cannot show whether dysbiosis causes lesion establishment, follows from gut physiology altered by chronic pelvic inflammation, or reflects shared upstream confounders such as diet, medication use or disease-associated behavior change. Prospective, ideally pre-diagnosis, sampling would be required to disentangle these. Such sampling is logistically difficult given the multi-year delay in Section 1, and to our knowledge no such cohort has been published. We use “dysbiosis” operationally: a reproducible, statistically supported difference in microbial community composition or inferred function between groups, not any single normative “healthy” configuration. Interpretation is further constrained by methodological heterogeneity: the sampling site (stool vs. vaginal, cervical, endometrial or peritoneal-fluid) and the sequencing approach (16S rRNA amplicon vs. shotgun metagenomic, with differing amplicon regions and pipelines). Others include small samples with limited correction for multiple testing, and low-biomass contamination in reproductive-tract and peritoneal specimens. Further confounding is unmeasured: menstrual-cycle phase, hormonal therapy, antibiotic, probiotic or analgesic exposure, bowel preparation, diet and irritable-bowel-syndrome comorbidity. Compositional findings from endometrioma, superficial peritoneal, deep infiltrating and adenomyotic phenotypes cannot therefore be assumed poolable. A 2025 systematic review and meta-analysis examined 11 observational (case–control/cohort) studies (1,727 women; 433 with endometriosis). It found modestly but significantly lower gut alpha-diversity in endometriosis by the Shannon (SMD 0.39, 95% CI 0.22–0.56) and Simpson (SMD 0.91, 95% CI 0.08–1.73) indices. The Chao1 index showed no significant difference (SMD 0.37, 95% CI −0.09–0.82). It also found qualitatively distinct beta-diversity and recurrent shifts such as a raised Firmicutes/Bacteroidetes ratio and depletion of Bifidobacterium and Lachnospira (26). The pooled evidence, entirely cross-sectional and heterogeneous (mixing 16S rRNA and shotgun methods with non-standardized diversity reporting), establishes association, not causation. It is best read as correlational support consistent with—but not proof of—the graded microbiota–estrobolome pathway advanced here.

Figure 2 summarizes the full dietary axis developed across this section, from food-group-level risk (3.1) through this mechanistic bridge (3.2) to specific therapeutic and probiotic interventions (3.3–3.5).

**Figure 2:**
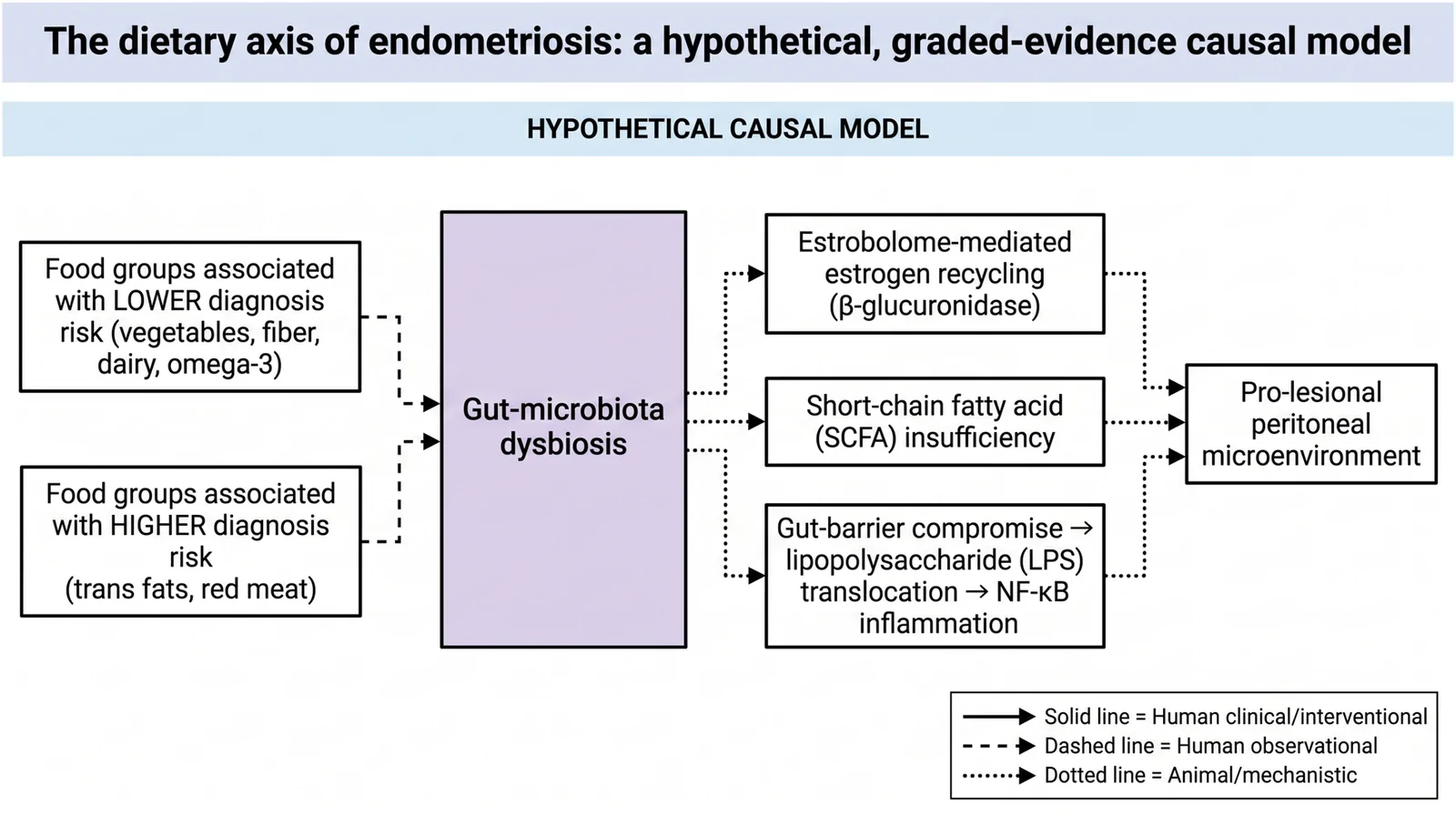
The dietary axis of endometriosis: a hypothetical, graded-evidence causal model. Food groups associated with a lower or higher risk of receiving an endometriosis diagnosis are hypothesised to act through gut-microbiota dysbiosis on three convergent mechanistic arms—estrobolome-mediated estrogen recycling, short-chain fatty acid (SCFA) insufficiency, and gut-barrier compromise with lipopolysaccharide (LPS) translocation—each of which is in turn hypothesised to contribute to a pro-lesional peritoneal microenvironment. Link style denotes the strength of the underlying evidence (solid, human clinical or interventional—no step shown here currently meets that standard; dashed, human observational; dotted, mechanistic hypothesis derived from animal models). Sources for the principal links: vegetable, cheese and dairy associations, Neri et al. (17); trans-fat and long-chain omega-3 associations, Missmer et al. (18); red meat, Yamamoto et al. (19); compositional dysbiosis, Yuanyue et al. (26) and Facciotti et al. (30); estrobolome concept, Salliss et al. (31); SCFA-macrophage and translocation-NETosis mechanisms, Xu et al. (32) and Wu et al. (33), both rodent. Most steps are extrapolated from other inflammatory conditions and from animal models and should be read as a testable organizing hypothesis rather than an established causal pathway. FMT, fecal microbiota transplantation; NETosis, neutrophil extracellular trap formation; NF-κB, nuclear factor-κB; TLR4, Toll-like receptor 4. Created in BioRender. Szili, K. (2026) https://BioRender.com/jc3p8gu.

### 3.3 Specific nutrients and supplementation: vitamin D and omega-3

Despite patient and clinician interest, the trial evidence for individual micronutrient supplementation remains disappointing. The SAGE trial (34) was a double-blind, randomized, placebo-controlled study in adolescent girls and young women (12–25 years) with surgically confirmed endometriosis. It screened 147 and randomized 69 to vitamin D3 (2,000 IU/day; *n* = 27), fish oil/omega-3 (1,000 mg/day; *n* = 20), or placebo (*n* = 22) for six months. Contrary to hypothesis, vitamin D produced pain reduction of similar magnitude to placebo. The fish-oil arm achieved only about half the visual-analogue-scale pain reduction of the other two arms. The authors note this may reflect an unusually large, persistent placebo response rather than a true absence of biological effect. It nonetheless constitutes a formally null trial for both supplements against the pre-specified primary endpoint.

This null result sits within a broader, inconsistent vitamin D literature. The most recent critical review (7) found only four vitamin D RCTs in endometriosis, two positive and two not; with small samples, this split precludes confident recommendation. Observational work repeatedly reports lower circulating 25-hydroxyvitamin D in endometriosis and an association between deficiency and diagnosis (35, 36). Systematic reviews of vitamin D's pathophysiological, diagnostic and therapeutic roles reach mixed conclusions (37, 38). The association is equally consistent with reverse causation, since chronic pain, reduced outdoor activity and chronic illness all lower vitamin D. Omega-3 evidence beyond the SAGE trial is similarly thin. The PurFECT1 trial (39) was a designated pilot, not powered for clinical effect. A 2026 synthesis in Frontiers in Nutrition (40) concludes that biological plausibility outpaces confirmatory trial evidence. Preclinical efficacy for both agents in experimental endometriosis (41) illustrates the familiar gap between animal efficacy and null human trials. The pattern is consistent: strong mechanistic rationale, sustained patient and clinician interest, and a trial base of pilot-scale studies where null or inconsistent findings are statistically expected.

### 3.4 Therapeutic dietary protocols: gluten-free, low-FODMAP, and anti-inflammatory approaches

The most methodologically robust interventional dietary study to date, van Haaps et al. (42), was a prospective, non-randomized controlled study at the Amsterdam UMC Endometriosis Center. Sixty-two women were enrolled, all with surgically or radiologically confirmed endometriosis and a baseline visual-analogue pain score of at least 3 cm. They were allocated to a low-FODMAP diet (*n* = 22), a patient-derived endometriosis diet excluding gluten, dairy and other components (*n* = 21), or no intervention (*n* = 19). Each group had dietitian support for three months, then three months of self-management. At six months, both diet groups reported reduced pain and improved quality of life against baseline in 6 of 11 Endometriosis Health Profile-30 domains. Against the control group, they reported significantly less bloating and better quality of life in three of eleven domains. FODMAPs (fermentable oligo-, di-, mono-saccharides, and polyols) are the substrates most readily fermented by colonic bacteria, so restricting them reduces fermentation and thus gas production and luminal distension. This stands in direct tension with the short-chain fatty acid (SCFA) argument of Section 3.2: reduced fermentation also lowers SCFA output, the metabolites described there as anti-inflammatory. Low-FODMAP restriction is therefore a short-term, symptom-directed intervention. Its gastrointestinal benefit costs the fermentative capacity a fiber-rich pattern supports, and it should be time-limited, with structured reintroduction to the widest tolerated diet, not a long-term anti-inflammatory strategy. A benefit under it is thus evidence about gastrointestinal symptom generation, not that the gut microbiota-estrobolome pathway of Section 3.2 has been validated. This also explains why these approaches signal more consistently for gastrointestinal and quality-of-life outcomes than for gynecological pain: the diet's physiological target maps onto the former. Gastrointestinal outcomes should be reported separately from dysmenorrhea, non-cyclical pelvic pain, dyspareunia and any objective disease measure. In a companion cross-sectional survey by the same group, 90 of 211 respondents reported strict adherence to a self-managed endometriosis diet. These adherents scored higher across all six quality-of-life domains than the 121 non-adherent respondents (*p* from <0.001 to 0.043). However, this design cannot exclude that women with milder disease, or greater health engagement, simply sustain adherence more readily.

A further caution follows from the population studied. Irritable bowel syndrome and other functional gastrointestinal disorders occur at elevated frequency in endometriosis, and the low-FODMAP diet is itself an established treatment for irritable bowel syndrome. Benefit in an endometriosis cohort may reflect treatment of a coexisting gastrointestinal comorbidity, not any effect on endometriotic lesions, inflammation or estrogen metabolism. This cannot be quantified from published data. Neither van Haaps et al. (42) nor its companion survey reports the proportion of participants meeting diagnostic criteria for irritable bowel syndrome, and the scoping review of De Araugo et al. (43) does not extract irritable-bowel-syndrome status across its studies. Future trials should record irritable-bowel-syndrome status at baseline against explicit criteria, and analyze gastrointestinal and gynecological outcomes separately. Without that stratification, a positive trial cannot distinguish treating the bowel from treating the disease.

Several narrative and scoping syntheses address the broader gluten-free and dietary-exclusion literature. These include the critical narrative assessment of Brouns et al. (44), the systematic review of dietary interventions by Nirgianakis et al. (45), and the 2025 scoping review by De Araugo and colleagues (43). That review identified seven RCTs, two non-randomized controlled studies, and four uncontrolled studies of diet/supplement interventions, converging on a cautiously optimistic but evidence-limited conclusion. Low-FODMAP approaches appear to have the most consistent trial-level support for gastrointestinal symptom and quality-of-life improvement. By contrast, gluten-exclusion and broader “endometriosis diet” approaches rest more heavily on patient-reported and cross-sectional than randomized evidence.

The gluten-free literature shows how one methodologically limited study can disproportionately shape clinical practice and patient expectation absent confirmatory work. Marziali et al. (46) gave a gluten-free diet to 207 symptomatic women with endometriosis for twelve months, in a single-arm, pre-post design without a control group. They reported 75% with statistically significant symptom improvement, 25% with no change, and none worsening. It is frequently cited in clinical and patient-facing sources as supporting gluten avoidance. However, a 2024 critical appraisal cautions against this. Marziali et al. remains essentially the sole published interventional evidence for gluten-free diets in endometriosis, and its single-arm, uncontrolled design cannot exclude substantial placebo, regression-to-the-mean, or concurrent-care effects. The appraisal also notes the largest prospective cohort points the opposite way. Schwartz et al. (47) analyzed 24 years of follow-up from 81,961 Nurses’ Health Study II participants, including 3,810 women with laparoscopically confirmed endometriosis. Higher gluten intake was associated with lower rather than higher endometriosis risk in primary analysis, but this did not survive the authors' own sensitivity analyses, which they characterized as unlikely to reflect a true etiological effect. The appraisal concludes that current evidence is insufficient to recommend gluten-free diets for endometriosis. Instead it suggests general healthy-eating guidance (limiting trans-fatty acids, alcohol, red meat, and salt), consistent with the food-group and dietary-pattern evidence in Section 3.1. This is more evidence-proportionate than nutrient-specific exclusion diets, pending adequately controlled gluten-free trials. Remarkably, given how often gluten-free diets are recommended in patient-facing and clinical contexts, such trials do not yet exist.

Commentary on the van Haaps trial (48) raised legitimate concerns about the nutritional adequacy and psychological burden of restrictive dietary self-management, particularly absent formal clinical guidelines on diet composition. It also called for closer integration between endometriosis researchers, registered dietitians, and food-service/hospitality stakeholders (e.g., low-FODMAP menu labeling) to reduce the adherence burden, a major barrier across studies. Prolonged restriction carries specific costs: reduced dietary diversity and inadequate fiber intake, the latter with its own adverse consequences for microbial diversity and short-chain fatty acid production. It also risks shortfalls in calcium, iron and B-vitamin intake where dairy and cereal groups are curtailed, plus higher food costs, social restriction, and poor long-term adherence. In a population already prone to symptom-driven food avoidance, there is a non-trivial risk of disordered eating. Any clinical use of a low-FODMAP or other exclusion diet in endometriosis should be time-limited, symptom-targeted, supervised by a trained dietitian, and planned to include a reintroduction phase.

### 3.5 Probiotic and synbiotic intervention

Probiotic supplementation is the most direct clinical translation of the gut microbiota-estrobolome hypothesis of Section 3.2. The trial evidence, though sparse, is more encouraging than that for micronutrient supplementation. Even so, only three small trials exist, differing in product, population and mechanism, so it reads as an early clinical signal requiring confirmation, not a consistently positive body of evidence. Khodaverdi et al. (49) ran a pilot, randomized, placebo-controlled trial in 37 women with surgically and pathologically confirmed stage III–IV endometriosis. Participants were randomized to an oral Lactobacillus-based product [LactoFem(R)] or placebo for eight weeks, with pain assessed via visual-analogue scale at baseline, eight, and twelve weeks. Dysmenorrhea improved most, peaking at the eight-week assessment. The authors frame their pilot's effect-size estimates as suitable for powering a future, larger definitive RCT, not confirmatory evidence in themselves.

An earlier, larger trial by Itoh et al. (50) randomized 66 women with clinically diagnosed endometriosis (62 completing the protocol). Participants received either a tablet containing 100 mg of heat-killed *Lactobacillus gasseri* OLL2809 (*n* = 29) or placebo (*n* = 33) for twelve weeks. This heat-inactivated preparation is, strictly, a postbiotic/paraprobiotic rather than a probiotic, since probiotics require live organisms. Both visual-analogue pain scores and verbal-rating-scale dysmenorrhea scores improved significantly more in the active arm than placebo, with no adverse effects reported. However, the trial was far too small for this absence of events to count as evidence of safety. The same group advanced a mechanistic rationale from a companion murine model: this *L. gasseri* strain stimulates interleukin-12 production from splenocytes, activating natural-killer-cell-mediated clearance of ectopic endometrial tissue in the peritoneal cavity. This is conceptually convergent with the broader estrobolome/immune-modulation hypothesis. However, strain-specificity means the finding should not be generalized to probiotics as a class.

A third pilot trial extends this to a different strain and endpoint set. Jara et al. (51) randomized 37 women (32 analyzed) with ultrasound-diagnosed peritoneal endometriosis, all maintained on dienogest 2 mg/day. Participants received a daily oral capsule of Ligilactobacillus salivarius CECT 30,632 (approximately 10^9^ CFU) or matched placebo for sixteen weeks. Its pre-specified primary outcomes—vaginal microbiota composition and inflammatory markers—were null. Benefits were confined to secondary endpoints. These were a within-group improvement in disease-specific quality of life (EHP-30 total score 49.0–30.9, *p* = 0.003) and an approximately 50% fall in serum 17β-estradiol (105.9–57.6 pg/mL, *p* = 0.013). Placebo produced no corresponding change, and the strain was well tolerated. These findings are hypothesis-generating at most: an unpowered pilot in already-diagnosed, pharmacologically-treated women, positive only on subjective secondary outcomes and—as set out below—at high risk of bias.

A randomized, double-blinded, placebo-controlled cross-over trial of a multi-species probiotic (ProMetrioS) has been registered and its protocol published (52). However, it has reported no outcome data and contributes nothing to efficacy conclusions at present; its completion should be monitored for any future update. A separate double-blinded RCT reported reduced menstrual pain with probiotic supplementation in primary dysmenorrhea (53). Because that population was not confirmed to have endometriosis, we do not count it as endometriosis-specific evidence, noting only that the two conditions share symptomatic and plausibly some mechanistic ground, so the result is hypothesis-generating at most.

We appraised risk of bias in the three completed endometriosis-specific probiotic trials with the Cochrane RoB 2 tool (Table 3). None reached low overall risk of bias: Itoh et al. (50) was rated “some concerns”, while Khodaverdi et al. (49) and Jara et al. (51) were both rated “high”. In each case this was driven by post-randomization exclusions analyzed without intention-to-treat. For Jara et al., it was additionally driven by retrospective trial registration and the foregrounding of secondary outcomes after null primary endpoints. Two of the three products were, moreover, supplied or funded by their manufacturers. This appraisal reinforces rather than tempers the cautious reading above: the probiotic evidence in endometriosis is a small, heterogeneous, bias-prone early signal, not a basis for any clinical preventive recommendation.

**Table 3:** Risk-of-bias assessment (cochrane RoB 2) of the three completed endometriosis-specific probiotic randomized controlled trials.

Trial	D1 Randomization	D2 Deviations	D3 Missing data	D4 Measurement	D5 Selection	Overall
Khodaverdi et al. (49)—LactoFem (live Lactobacillus)	Some concerns	Some concerns	High	Low	Some concerns	High
Itoh et al. (50)—heat-killed L. gasseri OLL2809 (postbiotic)	Some concerns	Some concerns	Some concerns	Low	Some concerns	Some concerns
Jara et al. (51)—L. salivarius CECT 30,632 (adjunct to dienogest)	Some concerns	Low	High	Some concerns	High	High

Assessed with the Cochrane Risk-of-Bias tool for randomized trials (RoB 2).

Each domain and the overall judgement are rated low risk, some concerns, or high risk of bias. No trial reached low overall risk of bias: Itoh et al. (50) was rated some concerns, and Khodaverdi et al. (49) and Jara et al. (51) high—driven principally by post-randomization exclusions analyzed without intention-to-treat and, for Jara et al., by retrospective trial registration and the foregrounding of secondary outcomes after null primary endpoints. Two of the three products were supplied or funded by their manufacturers. This appraisal underpins the low-to-very-low GRADE certainty assigned to the probiotic conclusion in Table 2.

Domains: D1, bias arising from the randomization process; D2, bias due to deviations from intended interventions; D3, bias due to missing outcome data; D4, bias in measurement of the outcome; D5, bias in selection of the reported result.

Beyond these three human trials, a supporting mechanistic literature (54) is relevant. It describes convergent routes by which probiotic strains might act beyond the estrobolome/β-glucuronidase pathway of Section 3.2: competitive exclusion of pathobionts, reinforcement of epithelial tight-junction integrity, and direct immunomodulatory signaling of the kind demonstrated for L. gasseri OLL2809. This plurality complicates trial interpretation as much as it encourages. Different strains are unlikely to share mechanisms, so a null result for one strain or product cannot be generalized to probiotics as a class. This caution applies to the antioxidant and anti-inflammatory literature of Section 3.6, where compounds as heterogeneous as vitamin C, resveratrol and allicin are routinely pooled.

### 3.6 Positioning relative to recent randomized-trial syntheses (2024–2026)

Between October 2024 and April 2026, six systematic or critical reviews quantitatively synthesized RCT evidence on dietary, nutraceutical or supplement interventions for endometriosis symptoms. These joined a substantial narrative literature since 2021 (55–57). Meneghetti et al. (6) screened 866 records across seven databases and meta-analyzed six RCTs (457 women). Pooled antioxidant supplementation reduced dysmenorrhea (mean difference −1.95, 95% CI −3.78 to −0.13), but not chronic pelvic pain or dyspareunia. Heterogeneity was high and results were null in trials at low risk of bias, which the authors read as evidence that reported benefits may be substantially attributable to bias. Wójtowicz et al. (7) extended this to 34 studies (23 RCTs) with a per-compound scorecard. There were single-study positives for selenium, propolis and quercetin, and an even split for vitamin D (two of four RCTs) and probiotics (one of two). Single-study findings for vitamin C, magnesium, resveratrol, N-acetylcysteine and palmitoylethanolamide-polydatin were unconfirmed. There was no RCT evidence for vitamin E, zinc, alpha-lipoic acid, epigallocatechin gallate or diindolylmethane. Four further reviews addressed narrower categories: antioxidants (58), anti-inflammatory supplements (59), vitamin C and E (60), and a scoping synthesis of the wider nutrition-intervention literature (43).

This body of work leaves little room for a further RCT-counting synthesis; rather than duplicate it, the present review makes two distinguishing contributions. First, it embeds the already-synthesized RCT evidence within the gut microbiota-estrobolome framework (Section 3.2) that none of the six reviews adopts. This reframing helps explain their heterogeneity, since interventions with a plausible upstream microbiome/estrogen mechanism (e.g., probiotics) show more coherent effects than those selected for generic antioxidant properties. Second, it situates dietary intervention within a broader multidimensional prevention model spanning risk modification, early screening, and cost-effectiveness—a scope entirely outside the treatment-phase focus of existing reviews. For a clinical supplement-by-supplement scorecard, the Wójtowicz et al. (7) per-compound synthesis remains the most current single reference; the present review explains, mechanistically, why that scorecard looks as it does.

### 3.7 The gut microbiome and fecal microbiota transplantation: mechanistic rationale, emerging evidence, and testable hypotheses

The dietary axis of Sections 3.1–3.6 converges mechanistically on one downstream effector: the gut microbiome. Diet may substantially modify endometriosis risk and symptom burden through microbial intermediaries—the estrobolome, short-chain fatty acid production, and gut-barrier integrity. If so, the microbiome becomes a candidate therapeutic target, and diet one route to it. This section summarizes recent controlled evidence on microbiome-directed intervention (including fecal microbiota transplantation, FMT) and sets out falsifiable hypotheses and trial designs to test them. The same estrobolome and SCFA biology explaining why diet matters also explains why direct microbial manipulation is investigated—the frontier of the dietary axis, not a separate topic.

#### 3.7.1 Human observational evidence: consistent dysbiosis, inconsistent taxa

Human studies detect compositional differences between the gut and reproductive-tract microbiomes of women with and without endometriosis, but disagree on which taxa drive the signal. The largest case-control analysis is a 1,000-woman subsample of the Estonian Microbiome cohort (136 with endometriosis) (61). It found distinguishing features but, like predecessors, modest effect sizes not replicating earlier candidate taxa. A 2025 scoping review attributes much disagreement to the methodological variation catalogued in Section 3.2 rather than an absent signal. Thus reproducible group-level differences in community structure are repeatedly observed, yet do not define a disease-specific state, and no single diagnostic or therapeutic taxon is validated. This favors function-level over taxon-level targeting and demands standardized methodology before trusting biomarker claims.

At the taxon level, several reports recur, though none is universal: a reduced Firmicutes-to-Bacteroidetes balance, depletion of putatively beneficial genera such as Lactobacillus in the reproductive tract, and enrichment of Gram-negative taxa contributing to lipopolysaccharide load. This fits the “bacterial contamination” hypothesis, in which increased endotoxin burden amplifies peritoneal inflammation (62). However, contemporary narrative and systematic reviews are explicit on two points. Individual markers such as Lactobacillus abundance are not reproducibly validated as diagnostic across cohorts. And the field's most reliable observation is a difference in community structure and inferred function rather than any named organism (30, 63). This matters for prevention: a robust functional dysbiosis is actionable through diet and metabolite-targeted intervention even when the responsible taxa remain unresolved.

The estrobolome, defined in Section 3.2, is the most therapeutically legible node of this axis. β-glucuronidase activity is measurable and diet-modifiable, linking a concrete enzymatic step to estrogen physiology clinicians already manipulate. A 2021 Human Reproduction Update synthesis placed this estrobolome-estrogen-inflammation triad at the center of the microbiome-endometriosis hypothesis (31). Studies since 2023 report altered estrobolome and immunomodulatory profiles in reproductive-age women with endometriosis. It anchors Hypothesis 1 below. Figure 3 depicts the enterohepatic estrogen-recycling loop and the readouts it implies.

**Figure 3:**
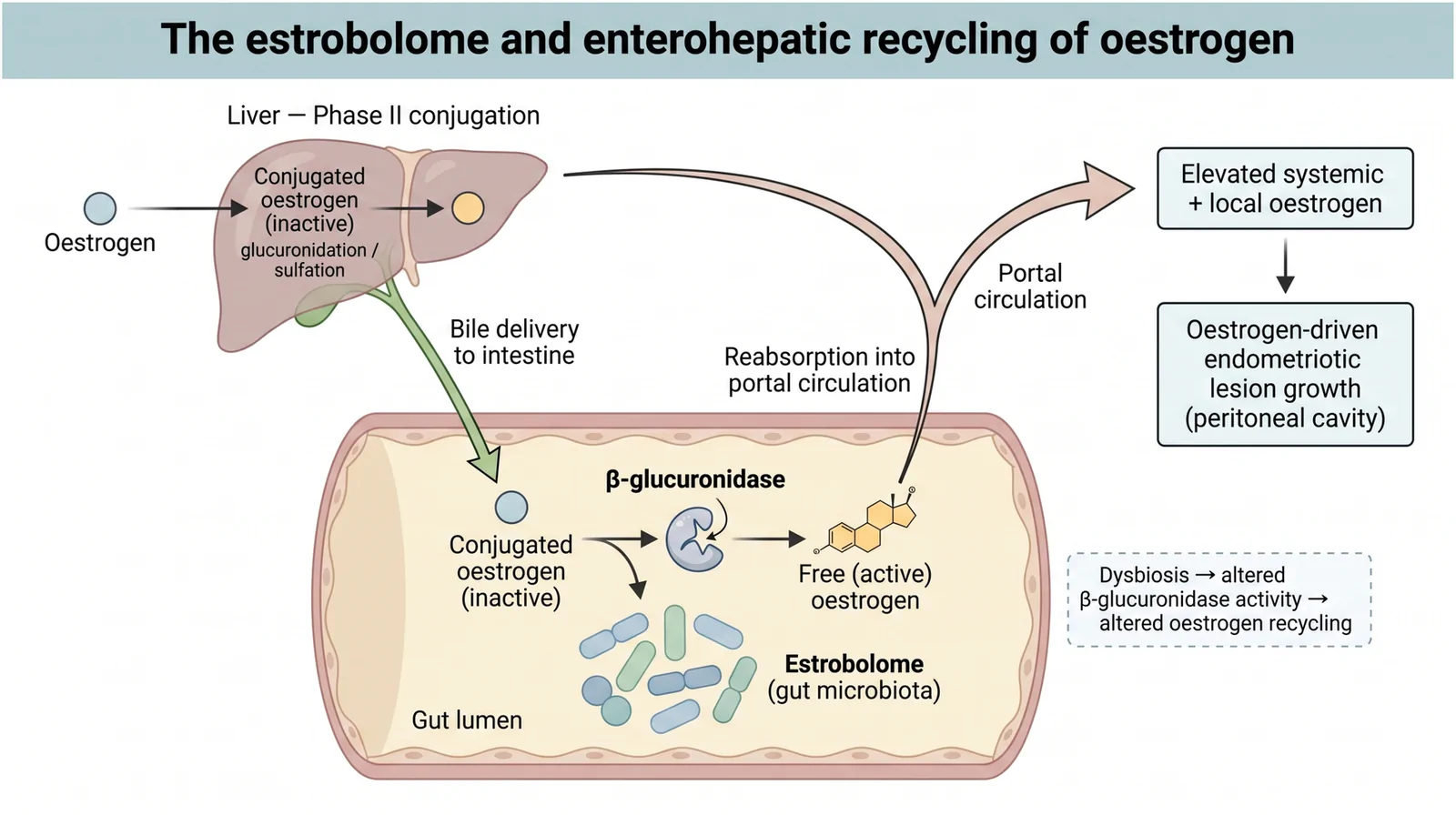
The estrobolome and enterohepatic estrogen recycling. Hepatically conjugated (inactive) estrogens excreted in bile are deconjugated in the gut lumen by bacterial β-glucuronidase, and the liberated bioactive estrogen is reabsorbed into the enterohepatic circulation, augmenting the estrogenic drive on ectopic lesions (31). Because β-glucuronidase activity is diet-modifiable and directly measurable, it is the most therapeutically legible node of the diet-microbiome axis (Hypothesis 1, Section 3.7.4). The whole pathway is presented as a mechanistic hypothesis: no step shown has been demonstrated in human endometriotic tissue, and most are extrapolated from other conditions and from animal models. Created in BioRender. Szili, K. (2026) https://BioRender.com/9bgn8lm.

#### 3.7.2 Causal and mechanistic evidence from animal models

Whereas human data are correlational, animal models currently ground causal claims along two threads. First, gut-microbiota-derived acetate ameliorated endometriosis in a mouse model via JAK1/STAT3-mediated M1 macrophage polarization. Fecal microbiota transplantation from healthy donors reduced ectopic lesion volume roughly four-fold vs. model controls (approximately 658 vs. 167 mm^3^), whereas endometriosis-donor transplantation did not (32). This donor-dependent, transferable phenotype is the strongest signal for a causal rather than correlative link. Second, intraperitoneal translocation of gut bacteria induced neutrophil extracellular trap formation that promoted endometriosis (33), a route by which a compromised gut barrier could seed pro-lesional inflammation. Together these motivate a testable chain, shown so far only in rodents: dysbiosis and barrier compromise reduce protective acetate signaling and increase bacterial or endotoxin translocation, shifting the peritoneal macrophage balance away from the acetate-driven, lesion-restraining M1 phenotype and toward a lesion-permissive M2 and NETosis-associated state, thereby facilitating lesion establishment. The chain is shown in rodents; the human steps are not.

#### 3.7.3 From mechanism to intervention: probiotics, FMT, and engineered approaches

The intervention literature spans three tiers of increasing ambition and decreasing maturity. Probiotic supplementation is most clinically advanced. Beyond the endometriosis-specific trials of Section 3.5, a 2026 randomized placebo-controlled trial reported reduced menstrual pain in primary dysmenorrhea (53). Fecal microbiota transplantation occupies the second tier, established in humans only for recurrent Clostridioides difficile infection. Proposed for gynecological indications on the strength of the rodent experiments above (64, 65), it lacks any controlled human FMT trial in endometriosis. The third tier—engineered consortia and next-generation probiotics selected for defined functions such as high butyrate output or low β-glucuronidase activity—remains preclinical. Any human FMT would confront the risks and governance already exposed in Clostridioides difficile practice. These include donor screening for transmissible pathogens, with documented and one fatal breach. They include evolving, jurisdiction-dependent regulatory classification and uncertain engraftment durability. Further concerns are possible transfer of undesirable metabolic, inflammatory or antimicrobial-resistance traits, and whether any microbiome change is causal or secondary. A final concern is the ethics of exposing stable patients to an invasive experimental intervention. FMT therefore belongs to a preclinical research agenda, not among candidate preventive interventions.

#### 3.7.4 Hypotheses and proposed solutions

On this basis we advance four falsifiable hypotheses, each with a study design. Hypothesis 1 (estrobolome-mediated estrogen recycling): endometriosis-associated dysbiosis elevates gut β-glucuronidase activity, increasing enterohepatic estrogen recirculation and sustaining a locally estrogenic milieu. It is testable by correlating fecal β-glucuronidase activity and serum unconjugated-estrogen fractions with disease stage. An interventional arm would use a dietary or pharmacologic β-glucuronidase modulator, with the unconjugated-estrogen fraction as primary endpoint. Hypothesis 2 (SCFA-dependent macrophage programming): microbiota-derived short-chain fatty acids shape peritoneal macrophage polarization, but the direction is metabolite-specific and, so far, demonstrated only in rodents; in a mouse fecal-microbiota-transplantation model higher gut acetate activated JAK1/STAT3 signaling and drove a protective M1 phenotype that reduced lesion volume, whereas acetate depletion favored the lesion-associated M2 phenotype and disease progression (32), while butyrate acts chiefly through histone-deacetylase inhibition and regulatory-T-cell promotion rather than through this M1 route. We therefore hypothesize that SCFA insufficiency removes these protective signals and shifts peritoneal macrophages toward the lesion-permissive M2 phenotype rather than producing an M1 skew, testable by a randomized fiber-based or direct-SCFA (e.g., tributyrin) intervention with paired fecal SCFA quantification and peritoneal-fluid M1/M2 profiling at surgery, analyzing acetate and butyrate arms separately. Hypothesis 3 (barrier translocation): increased intestinal permeability enables the bacterial translocation and NETosis shown to promote lesions in animals. It is testable by case-control comparison of barrier-integrity markers (zonulin, LPS-binding protein) and NETosis markers (citrullinated histone H3, cell-free DNA), with mediation analysis against severity. Hypothesis 4 (donor-dependent transferability): the therapeutic microbiome phenotype shown by donor-specific FMT in mice is reproducible in humans. It is testable only by a small, carefully governed phase-I/II trial, powered for safety and the biomarkers of Hypotheses 1–3, framed as hypothesis-generating rather than for a clinical endpoint.

These hypotheses imply a shift from taxon-hunting to function-targeting. The human taxonomic signal is heterogeneous, but the functional readouts (estrobolome activity, SCFA output, barrier integrity, peritoneal immune tone) are mechanistically coherent. The most tractable near-term program establishes these functional biomarkers as intermediate endpoints and validates them against surgical disease severity. Only then would they stratify patients and read out microbiome-directed interventions. Dietary intervention comes first, given its established safety and the evidence of Sections 3.1–3.6; FMT or engineered consortia come later, under trial conditions. This staged approach keeps the ambitious end (direct microbial therapeutics) tethered to the evidentiary standard the field meets. It positions diet not as a weak alternative but as microbiome manipulation's safest, best-supported first instrument. Figure 4 organizes this causal chain and the four hypotheses (H1–H4) with their biomarker endpoints and designs.

**Figure 4:**
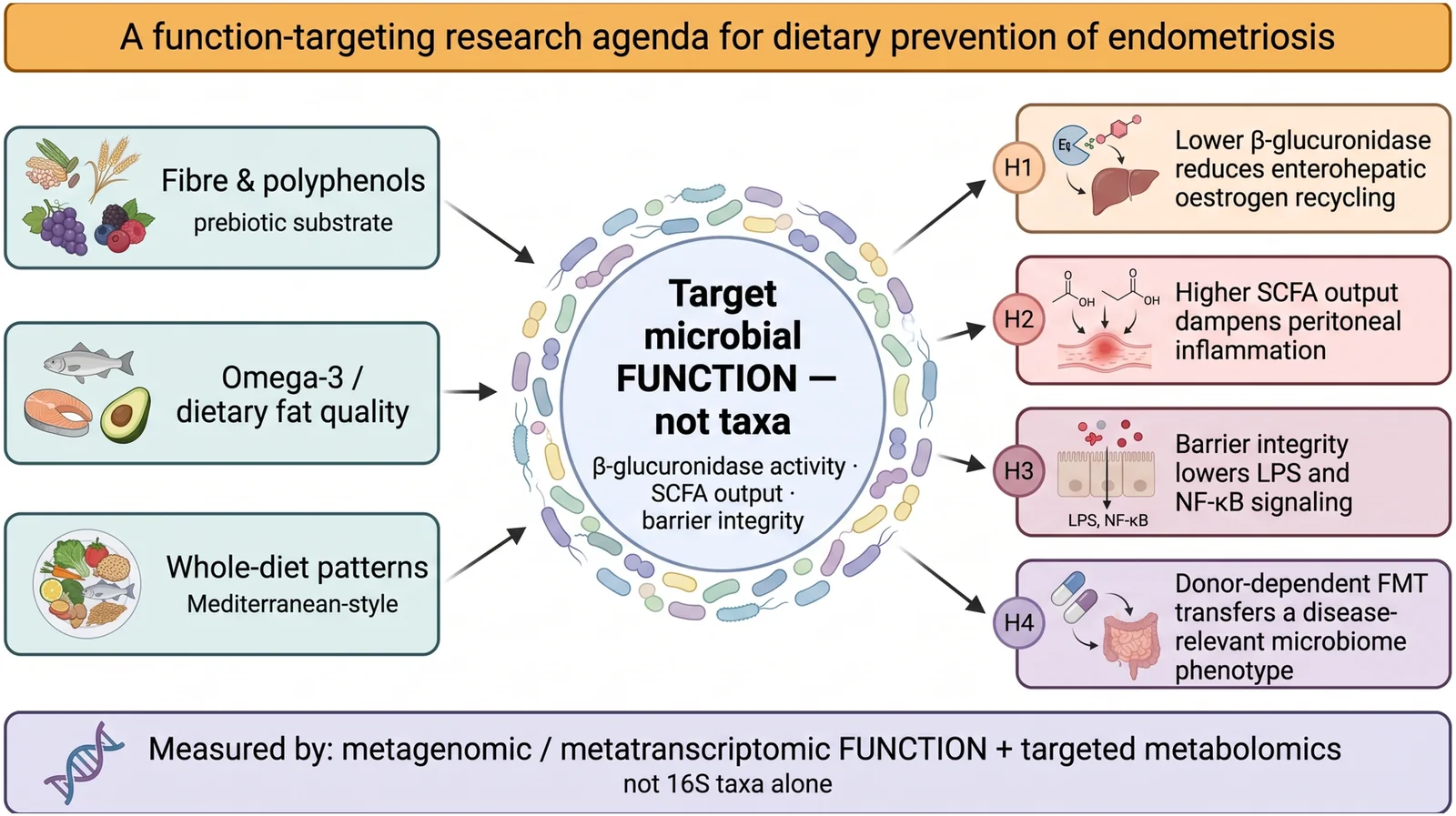
A function-targeting research agenda for the gut-endometriosis axis. Dietary inputs (fibre and polyphenols, omega-3 and dietary-fat quality, and whole-diet patterns) are proposed to act on microbial FUNCTION—β-glucuronidase activity, short-chain fatty acid output and gut-barrier integrity—rather than on specific taxa, motivating four falsifiable hypotheses (H1–H4), with the corresponding biomarker endpoints and study designs set out in Section 3.7.4; the underlying causal steps are demonstrated in rodent models only (32, 33) and the corresponding human steps have not been shown. Because the human taxonomic signal is heterogeneous whereas the functional read-outs are mechanistically coherent, the proposed strategy prioritizes validated functional biomarkers as intermediate endpoints, deploying dietary intervention first and reserving direct microbial manipulation (fecal microbiota transplantation, engineered consortia) for later evaluation under trial conditions. The description of diet as the first instrument to deploy refers to its comparative safety and to its being the best-supported of the microbiome-directed options considered here; as Section 8 states, no diet has been established to prevent endometriosis. LPS, lipopolysaccharide; SCFA, short-chain fatty acid. Created in BioRender. Szili, K. (2026) https://BioRender.com/dbot718.

#### 3.7.5 A non-invasive diagnostic corollary

The same functional readouts have a diagnostic corollary relevant to Section 5. Stool metabolite profiles differ in women with endometriosis. Because metabolites integrate community function, they may prove more reproducible than taxonomic counts. Recent work explicitly frames the microbiome as a source of non-invasive biomarkers (66). The validated readouts of estrobolome activity, SCFA output and barrier integrity are proposed in Section 3.7.4. If they also discriminate cases from symptomatic controls, they could serve as triage biomarkers. Like the therapeutic application, this needs no resolution of which taxa are causal, only a stable, measurable functional signature.

## 4 Primary prevention II: environmental endocrine disruptors

A further primary-prevention dimension is environmental endocrine-disrupting chemicals (EDCs)—dioxins and polychlorinated biphenyls, phthalates, bisphenols and pesticide residues—that interfere with estrogen-receptor signaling. A systematic review of non-persistent EDC exposure reported associations between urinary phthalate and phenol biomarkers and endometriosis (67). However, exposure-assessment heterogeneity severely limits causal inference, notably single-void urine capturing only recent exposure to short-half-life compounds. Broader syntheses conclude that endocrine disruption is mechanistically plausible on toxicological and animal-model grounds (68–70), but human epidemiological confirmation remains constrained by measurement error and near-ubiquitous background exposure. This supports precautionary population-level exposure reduction, not individual risk stratification. The EDC dimension also intersects the dietary axis: persistent lipophilic EDCs bioaccumulate in animal fat, so reducing red-meat and high-fat intake would plausibly lower dietary EDC exposure too. The association of total dairy with lower diagnosis risk (Section 3.1) nonetheless illustrates unresolved food-matrix complexity, visible only when the dietary and environmental literatures are read together.

## 5 Secondary prevention I: early non-invasive screening

**What screening does and does not mean in this review**

Earlier recognition and screening are not synonyms, and neither equals prevention. We distinguish seven activities this section and the wider literature frequently blend. Symptom awareness is the capacity of a young person, parent, teacher or clinician to recognize an abnormal symptom pattern; it is the target of the education programs in Section 5.3. Case finding is selective testing of individuals already presenting with relevant symptoms. Risk stratification assigns a pre-test probability from symptoms, family history or other markers to prioritize investigation. Diagnostic triage sorts symptomatic individuals toward or away from specialist referral and definitive investigation—what the tools in Sections 5.1 and 5.2 do. Screening, strictly used, is the systematic offer of a test to an asymptomatic population defined by age or other non-symptom criteria, within an organized program with defined invitation, quality-assurance and follow-up pathways. Diagnostic confirmation establishes the diagnosis; laparoscopy with histology remains the reference standard in many diagnostic-accuracy studies, but it is no longer universally required in current clinical practice, where a clinical and imaging-based diagnosis is increasingly accepted, including for deep and ovarian disease. Prevention of progression is a downstream clinical outcome, not a diagnostic activity.

On this taxonomy, none of the strategies below constitutes screening in the strict sense. A symptom checker used by someone who sought it out for symptoms is diagnostic triage of a self-selected symptomatic population. School-based menstrual-health education is symptom awareness. We retain “screening” in this section's title in its loose, established sense of earlier non-invasive detection, reserving the strict sense for organized programs. No organized endometriosis screening program exists in any health system, and we do not propose one.

The distinction matters because evidential requirements differ sharply. A triage instrument must report sensitivity and specificity against a stated reference standard. Because predictive values depend on prevalence, it must also report positive and negative predictive values for its actual target population. For a direct-to-consumer symptom checker, that is not the enriched specialist population in which such tools are usually developed. It must also quantify the consequences of error. False negatives give false reassurance and lengthen the delay the tool exists to shorten. False positives generate unnecessary imaging, referral, anxiety and, at the extreme, diagnostic laparoscopy. Extending any such tool to asymptomatic populations would additionally raise overdiagnosis—detection of disease that would never have produced symptoms or progressed. This cannot presently be excluded, since the natural history of untreated early lesions is not established, including the proportion that remain stable or regress. None of the tools below has been evaluated against this full set of requirements. The resulting harms are taken up in “Potential harms of early-detection strategies” below.

### 5.1 Non-invasive biomarkers

The multi-year diagnostic delay stems substantially from laparoscopy's role as the historical reference standard. Effort has therefore targeted non-invasive biomarkers to triage symptomatic women toward earlier referral. These include serum inflammatory/immune markers (including CA-125, limited in standalone sensitivity/specificity), microRNA signatures, and menstrual-effluent sampling, plus proteomic/metabolomic panels, with specific phospholipid species (71, 72). External validation in unselected symptomatic populations remains limited, and discovery-cohort performance often degrades on independent testing (“optimism bias”). Two reporting gaps recur and should be closed before any marker is proposed for triage. Sensitivity and specificity are typically derived from case-control designs contrasting confirmed cases with healthy controls, not the symptomatic women in whom the test would be used; this inflates apparent discrimination. Predictive values are rarely reported. Yet positive and negative predictive value, not sensitivity and specificity, determines what a result means for a patient at her setting's prevalence. The reference standard should also be stated, since studies verifying only test-positive women against laparoscopy incur partial verification bias. Reviews converge that no biomarker or panel yet achieves validated performance sufficient to replace laparoscopy or imaging. Combination approaches (biomarker plus symptom score plus imaging) outperform any single modality (73, 74). This mirrors diagnostic biomarker research in other chronic diseases.

### 5.2 Digital symptom checkers and structured triage

A parallel, more deployable strategy uses structured symptom-based digital triage tools. Rather than requiring novel biomarker discovery, they formalize existing symptom-pattern recognition into a scalable, low-cost instrument. Their intended use is specific: triage instruments for symptomatic individuals who have sought assessment, to shorten the interval to a first appropriate referral. They are not tests for asymptomatic populations, and they do not establish a diagnosis. Their published evaluations have not reported positive and negative predictive values in an unselected primary-care population. An economic evaluation published in 2026 (75) modeled such a checker's cost-effectiveness using a Markov decision-process framework; it is, to our knowledge, the only published endometriosis-specific economic evaluation of an early-detection intervention (Section 7.1). Their practical attraction is deployment to symptomatic individuals through primary care or consumer digital-health platforms. This addresses the interval between symptom onset and first specialist-relevant contact, which contributes most of the delay. The underlying clinical signal—a recognizable symptom cluster predating diagnosis—is reproducible enough to support such triage (76) while biomarker validation continues. Reviews of the primary-care approach and the primary-secondary care continuum both locate the principal bottleneck in the referral pathway itself, not test availability—precisely the segment these tools address (77, 78).

### 5.3 Adolescent menstrual-health education as population-level early recognition

Endometriosis frequently originates in adolescence yet is diagnosed years later. School- and community-based menstrual-health education therefore acts on the diagnostic interval. Part of that interval reflects adolescents—and the parents, teachers and clinicians they consult—normalizing severe dysmenorrhea; education targeting that normalization intervenes on the delay itself. A New Zealand school program improved recognition of symptoms warranting evaluation (79). Surveys document persistent gaps in adolescent and parental symptom literacy (80). Case-control work concludes adolescent dysmenorrhea warrants more structured investigation than current practice affords (81). Narrative reviews of endometriosis in adolescence converge on two observations: severe dysmenorrhea is routinely normalized, and prolonged undiagnosed disease carries a substantial psychological burden (82, 83). A 2024 cluster-randomized trial (84) provides the most rigorous evidence that structured education shifts symptom-literacy scores. However, translating knowledge gains into measurably earlier diagnosis requires longer follow-up than any study has achieved. This strategy remains underutilized relative to its plausible impact, although, as Section 7.1 sets out, its implementation cost is not negligible.

**Potential harms of early-detection strategies**

Earlier recognition's benefits must be weighed against potential harms, particularly where population-level or adolescent implementation is proposed. These include medicalization of common menstrual symptoms; false-positive risk-labeling in adolescents; and unnecessary imaging, specialist referral or laparoscopy. Further harms are initiation of long-term hormonal treatment without a secure diagnosis; treatment adverse effects and discontinuation; anxiety and altered reproductive expectations; and opportunity costs for health systems. We therefore caution against population screening of asymptomatic adolescents and long-term hormonal treatment on a presumptive diagnosis. We regard early hormonal interception (Section 6) as appropriate only where the diagnosis is established. A balanced framework must weigh both benefits and harms, not benefits alone.

## 6 Secondary prevention II: targeted early interception of established early-stage disease

Vercellini and colleagues' two-part proposal is the most developed framework for intercepting early-onset disease before progression to advanced-stage, more treatment-refractory disease. Part I articulates the pathogenic rationale. Early-onset endometriosis and adenomyosis are hypothesized to share upstream developmental and hormonal-exposure origins. Early intervention—before sustained cyclical bleeding, inflammation, and fibrotic remodeling establish a self-perpetuating disease process—may alter long-term trajectory as intervention after established, advanced-stage disease cannot (4). Part II translates this into candidate medical strategies, principally early hormonal suppression in adolescents and young women with a compatible symptom profile. It is positioned as secondary rather than primary prevention, depending on early symptom recognition (Section 5) as its entry point (5).

As the model is a proposal, not a validated pathway, its parameters need explicit statement, and where undefined we say so. The intended population is adolescents and young women with an established early-stage diagnosis—surgically confirmed, or supported by validated imaging—and a compatible symptom profile. Intervention on a presumptive diagnosis is not justified by current evidence, for the reasons under “Potential harms of early-detection strategies” above. The candidate interventions are ovulation- and menstruation-suppressing hormonal therapies already in routine use, principally combined oral contraceptives and progestins. Outcomes demonstrating disease modification rather than symptom suppression are progression to deep infiltrating or fibrotic disease, preserved fertility, and recurrence after treatment; none has been shown prospectively for early hormonal interception. Treatment duration is unspecified, as is the effect after discontinuation. This is critical, since a therapy suppressing lesion activity only while taken modifies symptoms rather than natural history. Long-term adherence and the adverse-effect burden of continuous hormonal therapy across the reproductive years, weighed against an as-yet-unquantified preventive benefit, are likewise unaddressed. The model has not been prospectively validated and no trial designed to test it has reported. Early hormonal treatment may be clinically reasonable for symptom control on its own merits—a separate, far better supported proposition than the claim that it alters long-term disease trajectory.

Applying the classification rule of “Defining prevention in this review”, we place this framework in the secondary-prevention tier, as its authors do, not the tertiary tier. It does not prevent initial lesion establishment (Sections 2–4). Although not itself a detection tool (Section 5), it acts on disease identified early through such tools. It is the early-intervention arm of a tier coupling early detection with early treatment, aiming to prevent progression to more severe, more treatment-resistant, and more fertility-compromising stages. This places it at the secondary-tertiary boundary, since preventing progression in diagnosed disease is also a tertiary objective. We resolve it in favor of the secondary tier because the intervention is defined by earliness of detection, not management of established disease. This arm's explicit dependence on the early-detection infrastructure above underscores why the model's four dimensions form an integrated system, not independent, competing research priorities. Without improved early symptom recognition, even a well-validated protocol has no practical entry point into clinical care.

The adenomyosis component warrants a separate note. Adenomyosis shares risk factors and hormonal dependence with endometriosis, and plausibly some dietary and microbiome mechanisms of Section 3, which is why the neo-evolutionary framework addresses both jointly. The primary-prevention strategies of Sections 2–4 may generalize to it in part. The evidence base is thinner still, however. The shared pathogenic account is inferred from those overlaps rather than demonstrated. We are aware of no prospective study testing whether early hormonal intervention alters the adenomyosis trajectory, and no dedicated adenomyosis-specific dietary or lifestyle intervention study has been published.

Translating this model into routine care would require a referral pathway most health systems lack: a mechanism for routing adolescents and young women identified through Section 5's strategies into early, protocol-driven management before advanced disease develops. The model has been articulated (4), yet such a pathway is absent—itself an illustration of the implementation gap this review highlights.

## 7 Cost-effectiveness as a cross-cutting dimension

### 7.1 The current endometriosis-specific evidence base

Endometriosis' economic burden is substantial and repeatedly quantified. United States estimates alone reach tens of billions of dollars annually combining direct medical, indirect productivity and informal caregiving costs. The cost-of-illness and treatment-comparison literatures are mature, spanning several health systems and covering surgical vs. medical management and newer GnRH-antagonist formulations. Essentially none of it has been extended upstream to prevention or early detection. To our knowledge, the 2026 Markov decision-process evaluation of a digital symptom checker is the only published endometriosis-specific cost-effectiveness analysis of a screening or early-detection intervention. It reports a favorable incremental net monetary benefit for digital triage in its base case. Its generalizability across health systems (which differ in baseline diagnostic delay and relative cost of primary vs. specialist care) is not independently confirmed. As a decision-analytic model, its effectiveness inputs are modeled assumptions, not prospectively observed real-world data. Two further limitations bear on interpretation. First, the intervention evaluated is a specific commercial menstrual-health application, not digital triage in general. Six of its ten authors declare an affiliation with the developing company, so it should be weighed alongside that paper's funding and competing-interest declarations. Second, the model has not been independently replicated. No dietary, lifestyle or probiotic intervention from Sections 2–3 has undergone a formal endometriosis-specific cost-effectiveness or cost-utility analysis. No adolescent education program from Section 5.3 has been economically evaluated using standard methods, even though its low per-pupil marginal cost would predict a favorable profile if tested. We correct here the framing used in the submitted manuscript: a low marginal cost per additional pupil is not a near-zero program cost. At scale, several further costs are real and attributable. These include curriculum development and revision, teacher and school-nurse training, clinician time, and digital infrastructure and content maintenance. They also include establishing and staffing a referral pathway able to absorb the additional presentations the program generates, quality assurance and evaluation, and the downstream diagnostic and treatment costs of that increased demand. Several fall on budgets other than the one that would realize the savings—itself an implementation barrier and an argument for the cycle-spanning cost accounting proposed in Section 7.4.

### 7.2 Methodological templates from analogous chronic-disease prevention economics

Absent endometriosis-specific evidence, the economic-evaluation methodology developed for lifestyle-intervention prevention in other chronic, diet-modifiable conditions—principally type 2 diabetes and cardiovascular disease—offers an adaptable template. Published evaluations of structured diabetes-prevention programs, including trial-based analyses, the Dutch SLIMMER program, and Markov comparisons of low- vs. high-intensity variants, provide worked examples. They model the long-term cost offsets of a low-cost behavioral intervention against the downstream costs of disease progression. Structurally the parallel holds: a modifiable upstream exposure (diet and lifestyle, or delayed diagnosis), a well-characterized downstream cost trajectory if unmodified, and a low-cost candidate intervention amenable to large-scale delivery. Substituting endometriosis-specific transition probabilities, costs and utility weights for their diabetes equivalents is, in our assessment, the most tractable route to the prevention economic evidence the field lacks.

### 7.3 A worked conceptual illustration: adapting the diabetes-prevention markov template to adolescent screening

To illustrate, we specify—as a template, not a result—how a school-based menstrual-health education program (Section 5.3) could be evaluated. Population: a hypothetical cohort of girls entering secondary education, modeled from age 12. Comparator: the usual curriculum, without structured menstrual-health education. Perspective: health-system and education-sector costs combined, with a societal perspective (productivity, informal care) secondarily, since costs and savings accrue to different budgets. Time horizon: to age 50, long enough to capture fertility-related outcomes. Structure: a Markov cohort model with an annual cycle and the health states “no symptoms”, “symptomatic, undiagnosed”, “diagnosed, medically managed”, “diagnosed, surgically managed”, “advanced or recurrent disease”, and death from background causes. Costs: primary-care and specialist contacts, imaging, laparoscopy, medical and surgical treatment and assisted reproduction, taken from national tariff or reimbursement sources and discounted at the guideline-specified rate, with the same rate applied to health outcomes. Utilities: health-state utility weights from published endometriosis quality-of-life studies, the decrement attached to the “symptomatic, undiagnosed” state being a principal driver. Analysis: incremental cost per quality-adjusted life-year, with one-way deterministic sensitivity analysis across the plausible diagnostic-delay range and probabilistic sensitivity analysis over all parameters.

Separating what could be parameterized from what could not is the point of the exercise. Cost inputs and utility weights could largely be drawn from published sources today. Background transition probabilities between the symptomatic-undiagnosed and diagnosed states could be approximated from health-system diagnostic-pathway data, with acknowledged imprecision. The intervention effect could not. We retract the suggestion, in the submitted manuscript, that such analysis could be performed without new primary data. The mean or median diagnostic delay is a descriptive interval, not a transition probability, and cannot be converted into one. Moreover, the effect of education on diagnosis timing—let alone its onward effects on progression, treatment utilization, fertility and quality-adjusted life-years—has not been estimated in any study. The best available evidence (84) demonstrates a change in knowledge scores, not time to diagnosis. The model structure can be specified now, as above. Its central effectiveness parameter, however, requires a prospective study linking an education program to measured time-to-diagnosis before yielding a dependable result. That study, not the model, is the immediately actionable item in this review's agenda.

### 7.4 Toward a value-based care model: an integrating proposal

The four dimensions share a structural feature a value-based care framework makes explicit. Each is justified less by its isolated effect than by health outcomes delivered per unit cost across the full care cycle, from at-risk adolescence to established disease (85). So reframed, the model is a candidate value pathway, not a taxonomy. It has three layers: a mechanistically grounded dietary and microbiome-targeted primary layer (Sections 3–4), a low-marginal-cost early-detection layer compressing the diagnostic interval (Section 5), and a protocol-driven early-intervention layer (Section 6). Each serves the few outcomes that matter to patients—pain and quality of life, preserved fertility, time to diagnosis—not isolated process metrics.

Operationalizing it requires three commitments the evidence base cannot yet support but could generate. First, a defined outcome set measured longitudinally across the care cycle, not single encounters. Second, cost accounting over the same cycle and payers, so upstream interventions and downstream offsets fall to one ledger. That ledger is what the diabetes- and cardiovascular-prevention evaluations were built to capture (86–90). The single endometriosis-specific evaluation begins to model it too (75). Third, an implementation pathway assigning explicit responsibility for the referral mechanism most health systems lack (Section 6).

We advance this as a proposal, not a validated model. Its central claim is testable: bundling the four dimensions into one outcome- and cost-accountable pathway would deliver better patient-relevant outcomes per unit cost than the fragmented, treatment-weighted status quo. The first step is outlined in Section 7.3.

## 8 Discussion

This review set out to integrate four dimensions of endometriosis prevention into one explanatory framework. These are primary risk-factor modification (emphasizing a mechanistically grounded dietary/microbiome axis), secondary prevention through earlier detection and targeted early intervention, tertiary-level management of established disease, and cross-cutting cost-effectiveness evaluation. The integration is motivated by their being typically studied, and published, in isolation.

Controversies and unresolved questions. The dietary and nutraceutical trial literature shows small samples, moderate-to-high risk of bias and high heterogeneity. Most tellingly, trials at low risk of bias tend toward null results, whereas higher-risk trials show benefit (6). That is a recognized signature of publication bias, small-study effects, or inadequate blinding in hard-to-blind interventions. It counsels against premature enthusiasm for any single dietary or supplement approach. Once reported, ProMetrioS will be an important test case. The gluten-free controversy of Section 3.4 is its acute form: a widely repeated, patient-facing recommendation on one uncontrolled study, apparently contradicted by the largest prospective cohort. A second is whether population-wide primary prevention is a coherent goal at all. Modifiable risk factors so far carry effect sizes typically between 0.6 and 1.3, and a substantial heritable component caps any behavioral strategy. A third is measurement. In most observational studies, endometriosis risk means risk of diagnosed endometriosis, which depends on access, reporting and clinician suspicion. Part of every reported dietary association may therefore reflect differential care-seeking, not a biological effect on incidence. This limitation is intrinsic to the Section 3.1 literature and rarely explicit in the primary studies.

Foundational concepts: the case for an integrated model. The multidimensional framing's principal value is integration itself, not any single empirical finding. Dietary and probiotic interventions are more interpretable when tied to the gut microbiota-estrobolome hypothesis (Section 3.2) than as undifferentiated alternatives to hormonal therapy. Early hormonal interception (Section 6) is actionable only if earlier detection (Section 5) identifies eligible patients. The near-total absence of cost-effectiveness evidence (Section 7) applies across all three tiers, a shared methodological gap, not four unrelated shortfalls. Read in isolation, each domain looks promising yet insufficient. Read together, they are mutually reinforcing, and under-investment in any one—economic evaluation above all—constrains what the others achieve.

Research gaps. Four gaps stand out. First, essentially no endometriosis-specific cost-effectiveness or cost-utility analysis exists for any prevention or early-detection strategy. This contrasts with the mature cost-of-illness literature documenting the burden they would offset. Section 7.3 specifies one route to begin closing this gap, and what it still requires. Second, interventional probiotic research remains early-stage: three small completed trials, one unreported ongoing trial, and no strain-selection framework grounded in β-glucuronidase or estrobolome-modulating activity—despite being the most mechanistically coherent dietary avenue. Third, no prospective pre-diagnosis microbiome cohort in endometriosis has been published, which any causal claim about dysbiosis ultimately requires. Fourth, essentially none of the primary-prevention literature of Sections 2–4 comes from low- and middle-income-country populations. There, dietary patterns, background exposures and diagnostic capacity all differ from the North American and Western European cohorts that dominate the evidence base.

**Current implications for clinical practice**

Pending stronger evidence, a bounded set of statements is possible. A balanced, nutritionally adequate diet and regular physical activity are reasonable for general health. No specific diet has been established to prevent endometriosis. A low-FODMAP diet may help selected patients with gastrointestinal symptoms and should be time-limited and dietetically supervised. Routine gluten exclusion is not supported absent celiac disease, non-celiac gluten sensitivity or another established indication (91). Evidence for vitamin D, omega-3 and probiotic supplementation remains insufficient for universal recommendation. Fecal microbiota transplantation and engineered microbiome interventions remain experimental. Menstrual-health education and improved diagnostic pathways are promising but require evaluating benefits, harms and cost-effectiveness. Finally, evidence that early hormonal treatment modifies long-term disease progression remains incomplete.

## 9 Conclusion

Endometriosis prevention remains a largely unrealized opportunity, and the framework here is deliberately hypothesis-generating, not prescriptive. The strongest current signals are indirect: observational diet–diagnosis associations, a biologically coherent but human-unproven gut-microbiota–estrobolome pathway, and the promise of shortening diagnostic delay through non-invasive detection and menstrual-health education. By contrast, interventional evidence in diagnosed patients speaks to symptom management, not to preventing incident disease. Accordingly, no dietary, microbial, or screening strategy can yet be recommended for prevention outside research settings. What the evidence supports is a proportionate agenda. Prioritize the safe, low-cost measures reasonable on general-health grounds, subject the estrobolome and function-targeting hypotheses to the falsifiable tests set out above, and generate the endometriosis-specific cost-effectiveness data prevention policy will require. Judged against that standard, the field's task is not to over-promise prevention but to build, tier by tier, the evidence that makes prevention actionable. Future directions. Priority investments are fourfold. (i) Completion and mechanistic sub-analysis of ProMetrioS and comparable probiotic trials, with strain selection informed by estrobolome-modulating activity, not generic probiotic branding. (ii) A prospective study linking adolescent menstrual-health education to measured time-to-diagnosis; without it, the cost-utility analysis sketched in Section 7.3 cannot be parameterized. (iii) Larger, adequately blinded confirmatory trials with pre-registered outcomes for dietary and supplement interventions supported only by small or high-risk-of-bias studies. (iv) Extension of the primary-prevention literature to more geographically and socioeconomically diverse populations. We propose organizing these within the value-based framework of Section 7.4. This means a defined, patient-relevant outcome set measured across the full care cycle and matched to cycle-spanning cost accounting, with the adolescent-education analysis as its first empirical anchor.

### 9.1 Limitations

Limitations. Several limitations qualify this synthesis. First, it is a narrative, not systematic, review. For randomized-trial evidence, it relies on six systematic reviews, not independent primary screening. Selective citation and publication bias therefore cannot be fully excluded, and the pattern of low-risk-of-bias dietary trials tending toward null results (6) counsels caution. Second, the gut microbiota-estrobolome framework organizing the dietary axis is largely extrapolated from other inflammatory conditions and animal models. It has not been demonstrated in human endometriotic tissue and should be read as a testable hypothesis. Third, most primary evidence derives from high-income North American and Western European cohorts, limiting generalizability to lower-resource settings with differing dietary patterns, background exposures, and diagnostic capacity. Fourth, “endometriosis risk” in observational studies typically means risk of diagnosed endometriosis, so some reported dietary associations may partly reflect differential care-seeking or ascertainment, not true effects on incidence. Finally, several foundational primary studies predate the four-year window preferred for a strictly contemporary evidence base. These are retained only where no equivalent recent primary evidence exists, and flagged as such in the reference-validation table.
